# Spinal cord stimulation for Tarlov cyst-related pain: Initial success and subsequent explantation in an elderly patient

**DOI:** 10.1016/j.inpm.2025.100604

**Published:** 2025-06-21

**Authors:** Alexandre J. Bourcier, Christina Im, Jane Phan, Michelle Nwufo, Miad Hadaegh, Colton M. Malesovas, Jae Jung, Kyle Yang, Jonathan Droessler

**Affiliations:** a David Geffen School of Medicine at the University of California - Los Angeles, Los Angeles, CA, USA; b Department of Orthopaedic Surgery, University of California - Los Angeles, Los Angeles, CA, USA

Dear Editor

Tarlov cysts, or perineural cysts, are cerebrospinal fluid (CSF) –filled dilations most frequently arising from the sacral nerve root sheaths. Although frequently incidental, these lesions can produce severe neurologic symptoms when they enlarge or compress adjacent neural structures [1,2]. In rare cases, they lead to persistent radiculopathy, back pain, or pelvic dysfunction [1]. Treatment options are often limited by the risks of surgical intervention, particularly CSF leak, and by the lack of high-quality evidence supporting conservative or procedural approaches [[3], [4], [5]]. Spinal cord stimulation (SCS) has emerged as a potentially valuable modality for managing chronic neuropathic pain, though its application in Tarlov cyst-related pain remains sparsely described [6,7].

We present the case of a 77-year-old woman with a longstanding history of low back pain and right-sided leg radiculopathy attributed to a large sacral Tarlov cyst. MRI demonstrated an intraforaminal lesion at the right S1 nerve root causing neural compression (Fig. 1, Fig. 2). The patient had exhausted conservative management including neuropathic medications, physical therapy, and two fluoroscopically guided transforaminal epidural steroid injections. While she did achieve temporary anesthetic relief from the injections, long-term therapeutic benefit was absent. Given her age and comorbidities, surgical intervention was deferred due to high risk of CSF leak.

**Fig. 1:**
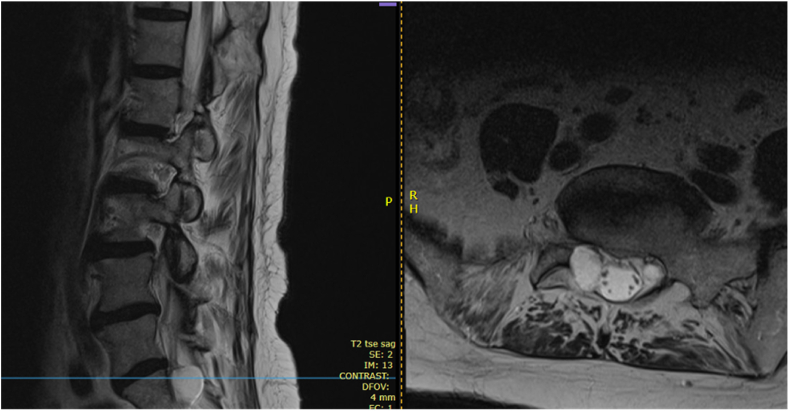
Sagittal and axial views of lumbosacral MRI demonstrating the superior portion of a Tarlov cyst at S1.

**Fig. 2:**
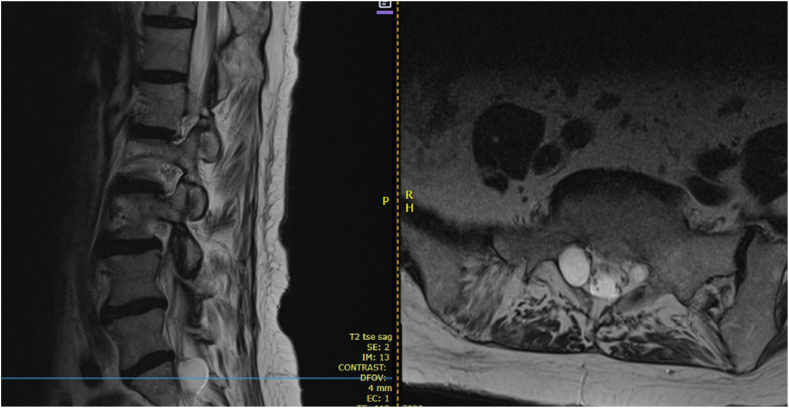
Sagittal and axial views of lumbosacral MRI demonstrating the inferior portion of a Tarlov cyst at S1.

In March 2024, the patient underwent a trial of SCS using percutaneous leads placed at the T8–T9 vertebral level. Sub-perception stimulation was applied over the region corresponding to her symptoms. During the trial, she reported approximately 70 % improvement in pain intensity and meaningful gains in walking distance and participation in daily activities. Radiographic confirmation demonstrated optimal lead placement with tip coverage at the superior aspect of the T10 vertebral body (Fig. 3). Based on these results, she proceeded with permanent implantation in April 2024 using a non-rechargeable pulse generator implanted in the right upper buttock.

**Fig. 3:**
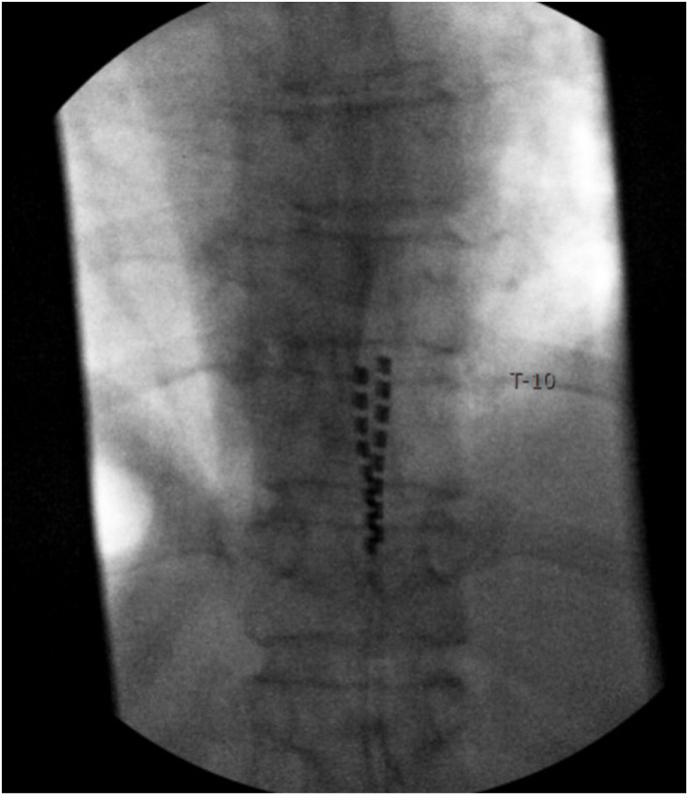
Fluoroscopic imaging demonstrating SCS lead placement in the epidural space with lead tips at the superior border of T10.

While her radicular symptoms remained well controlled post-implantation, she developed persistent pain localized to the implantable pulse generator (IPG) pocket. Reprogramming failed to mitigate this discomfort. In June 2024, she underwent surgical revision to relocate the pocket and replace the device with a smaller, rechargeable IPG. This intervention resolved her localized pain and maintained the therapeutic effect on her radicular symptoms.

At three- and six-month follow-ups, the patient remained off opioid medications and reported improved function with continued benefit from SCS. However, by her one-year follow-up in April 2025, she expressed growing dissatisfaction related not to symptom recurrence but due to her challenges in managing the device. She cited frustrations with charging, uncertainty about therapy activation, and diminished confidence in interacting with the system. Despite education reinforcement and support, she ultimately elected to undergo explantation due to a perceived decline in usability and utility.

This case illustrates both the therapeutic potential and the real-world limitations of SCS in older adults with complex pain syndromes. While multiple case reports have described SCS use in patients with Tarlov cyst-related pain, often after failed surgical management or in combination with other interventions, this case highlights a scenario in which neuromodulation was employed instead of surgery with initial success. The patient experienced substantial improvements in pain and functional outcomes, but later chose explantation due to perceived burden of device management.

A key limitation in many published reports is the narrow focus on short-term efficacy. SCS should not be evaluated solely by technical success or pain scores during trial or early post-implantation periods. Sustained benefit requires patients to engage meaningfully with their device over time. Our patient's experience draws attention to a broader spectrum of success metrics in neuromodulation, including cognitive and technological competence, support infrastructure, and evolving patient preferences.

The increasing reliance on rechargeable pulse generators offers theoretical benefits, such as smaller size and longer lifespan, but may inadvertently disadvantage patients with limited digital literacy, memory deficits, or poor caregiver support. While device rechargeability solves certain mechanical constraints, it introduces new behavioral demands. For some patients, especially older individuals, this can undermine the perceived value of the therapy over time.

This case also underscores the need for systematic patient selection criteria that go beyond anatomical targeting or pain phenotype. Incorporating assessments of cognitive function, health literacy, and technological skills may help identify patients who are more likely to benefit in the long term. Additionally, a structured post-implantation follow-up strategy, including patient education refreshers, check-ins on device use confidence, and reinforcement of therapeutic goals, may help reduce dropout or explantation in otherwise appropriate candidates.

From a therapeutic standpoint, this case adds to the emerging clinical discussion around neuromodulation for sacral cyst-related pain. Although not unprecedented, it contributes a year-long perspective on symptom relief, revision, and eventual discontinuation, reflecting the full arc of real-world neuromodulation therapy. These insights may guide interventional pain physicians in setting expectations and in designing care models that account for the behavioral dimensions of SCS.

In conclusion, spinal cord stimulation provided meaningful symptom relief for this patient with Tarlov cyst-associated radiculopathy, offering an alternative to high-risk surgical intervention. However, her eventual decision to explant the device illustrates the importance of aligning therapy with a patient's evolving capabilities, preferences, and support system. As the field continues to innovate, long-term success will depend not only on the hardware or waveform, but on how well the system integrates into the lives of those it aims to help.

## Patient consent

Informed consent was obtained from the patient for publication of this case report and any accompanying images.

## Declaration of generative AI and AI-assisted technologies in the writing process

During the preparation of this work the authors used ChatGPT in order to improve writing style and proofread for any inconsistencies. After using ChatGPT, the authors reviewed and edited the content as needed and take full responsibility for the content of the publication.

## Funding

This research did not receive any specific grant from funding agencies in the public, commercial, or not-for-profit sectors.

## Declaration of competing interest

The authors declare that they have no known competing financial interests or personal relationships that could have appeared to influence the work reported in this paper.
